# High-accuracy transmission and fluorescence XAFS of zinc at 10 K, 50 K, 100 K and 150 K using the hybrid technique

**DOI:** 10.1107/S1600577522010293

**Published:** 2023-01-01

**Authors:** Marcus W. John, Daniel Sier, Ruwini S. K. Ekanayake, Martin J. Schalken, Chanh Q. Tran, Bernt Johannessen, Martin D. de Jonge, Peter Kappen, Christopher T. Chantler

**Affiliations:** a School of Physics, University of Melbourne, Melbourne, Australia; b La Trobe University, La Trobe, Australia; c Australian Synchrotron, ANSTO, Clayton, Australia; University of Essex, United Kingdom

**Keywords:** XAFS, hybrid technique, transmission and fluorescence, zinc, thermal evolution

## Abstract

The most accurate measurements of the mass attenuation coefficient for metals at low temperature for the zinc *K*-edge from 9.5 keV to 11.5 keV at temperatures of 10 K, 50 K, 100 K and 150 K using the hybrid technique are reported. This is the first time transition metal X-ray absorption fine structure (XAFS) has been studied using the hybrid technique and at low temperatures. This is also the first hybrid-like experiment at the Australian Synchrotron. The measured transmission and fluorescence XAFS spectra are compared and benchmarked against each other with detailed systematic analyses. A recent method for modelling self-absorption in fluorescence has been adapted and applied to a solid sample. The XAFS spectra are analysed using *eFEFFIT* to provide a robust measurement of the evolution of nanostructure, including such properties as net thermal expansion and mean-square relative displacement. This work investigates crystal dynamics, nanostructural evolution and the results of using the Debye and Einstein models to determine atomic positions. Accuracies achieved, when compared with the literature, exceed those achieved by both relative and differential XAFS, and represent a state-of-the-art for future structural investigations. Bond length uncertainties are of the order of 20–40 fm.

## Abstract 2

High-accuracy measurement of the X-ray absorption fine structure (XAFS) of zinc metal in a temperature series from 10 K to 150 K, including 298 K, simultaneously in transmission and fluorescence using the hybrid technique is presented: the first transition metal XAFS using the hybrid technique and at low temperatures; and the first (hybrid-like) experiment at the Australian Synchrotron. A methodology for relative measurements and a methodology for cryostat measurements, and an approach to normalization, calibration of transmission measurements for scattering and other systematics to reference room-temperature data and yield a defined uncertainty are presented.

## 1. Introduction

X-ray absorption spectroscopy (XAS) has been a major field in physics for more than 100 years, and thousands of XAS papers are published each year. It enables investigation of widely used physical parameters such as the mass attenuation coefficient, atomic form factors and nanostructure in both solids and solutions. The development of the X-ray extended range technique (XERT) by Chantler *et al.* (2012*a* ▸) has enabled a greatly improved accuracy to be achieved in X-ray absorption near-edge structure (XANES) and extended X-ray absorption fine structure (EXAFS), with improvements often by orders of magnitude, and through this improvement discrepancies with theoretical predictions have been revealed. Despite the high dependence on XAS techniques in a wide range of scientific fields, very few experiments using standard X-ray absorption fine structure (XAFS) configurations have sufficient accuracy to test the method and current theoretical understanding. The development of the *hybrid technique* (Chantler *et al.*, 2012*b* ▸, 2015 ▸; Islam *et al.*, 2015 ▸, 2016 ▸; Schalken & Chantler, 2018 ▸; Trevorah *et al.*, 2019 ▸) is an attempt to overome the inadequacies of conventional XAFS systems. The hybrid technique allows for simultaneous measurement of transmission and fluorescence spectra. These are usually measured independently, but here can be directly compared. It has been found valuable for: dilute solutions including ferrocene and derivatives; for determining conformations when all shells of the different conformers are at the same radii; for nickel complex solutions; and for distinguishing local geometries where coordination numbers are identical. It has not, however, so far been applied to (solid or transition) metal foils. Also, it has not previously been applied to temperature series or low-temperature measurements.

Low-temperature measurements, usually with only a single small sample in the cryostat, also usually involve samples which cannot have their total surface profile mapped in the beam. A detailed two-dimensional *xy* scan is important to measure the local thickness in the beam and the integrated column density [ρ*t*] and hence for absolute measurement of the attenuation coefficient 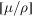 . This lends itself to the idea of using the hybrid technique and the use of some external reference (a secondary reference foil outside the cryostat which can be mapped), where normalization can proceed via transmission measurement. Here we use the same samples for fluorescence and transmission (*i.e.* internal and external references) to calibrate several possible systematics, in order to attain higher accuracy and insight.

Temperature series measurements, or the investigation of structural thermal evolution using XAS, has many great achievements, especially for the investigation of small differential displacements (Pettifer *et al.*, 2005 ▸; Ruffoni *et al.*, 2007 ▸) and for the evolution of distinct phases often at higher temperature or pressure (Araujo *et al.*, 2006 ▸; Brugger *et al.*, 2007 ▸; Testemale *et al.*, 2009 ▸; Giulian *et al.*, 2009 ▸). Some of the best early work on thermal evolution for single-phase spectra investigated the development of cumulant analysis (Greegor & Lytle, 1979 ▸; Sevillano *et al.*, 1979 ▸; Tröger *et al.*, 1994 ▸; Dalba & Fornasini, 1997 ▸; Fornasini *et al.*, 2004 ▸; Abd el All *et al.*, 2013 ▸).

Here we investigate the thermal evolution of zinc metal foil. Zinc is fairly well studied by XANES (Kraft *et al.*, 1996 ▸), though repeated studies have found energy discrepancies of 2 eV or so (Eisa *et al.*, 2005 ▸). A temperature series of X-ray diffraction (XRD) (Nuss *et al.*, 2010 ▸) has provided valuable data on changes of the separations of the lattice sites. Several studies have investigated XAFS around zinc sites in compounds or complex systems (Gilbert *et al.*, 2002 ▸; Chung *et al.*, 2000 ▸; Sier *et al.*, 2020 ▸). An XAFS study of zinc metal (Rae *et al.*, 2010 ▸) yielded too sparse a grid for detailed analysis, though a recent work at room temperature using XERT (Ekanayake *et al.*, 2021*a* ▸,*b* ▸; Sier *et al.*, 2022 ▸) laid a strong and detailed baseline from which thermal evolution could be investigated. This is important for reference nanostructure and cross-platform portability when comparing analysis from XERT and hybrid, fundamental studies of zinc, and also for studies of small-scale atomic displacements and thermal expansion, the robustness of XAFS software, and particularly the extraction and understanding of the inelastic mean free path of the photoelectron and associated plasmon structure (Bourke & Chantler, 2014 ▸).

## 2. Hybrid attenuation measurements: experimental

**Figure 1:**
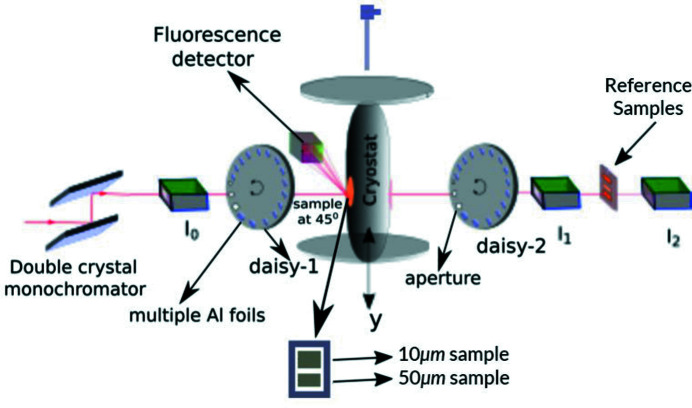
Experimental hybrid setup (Chantler *et al.*, 2012*b* ▸) at the Australian Synchrotron. The sample holder in the cryostat is offset vertically to select the appropriate sample in the beam path. *I*_0_ and *I*_1_ take attenuation measurements of the sample in the cryostat, while *I*_1_ and *I*_2_ measure reference foils (Islam *et al.*, 2016 ▸). Daisy wheels, on either side of the sample, characterize harmonic and scattering systematics (Tran *et al.*, 2004 ▸).

The experiment was performed at the Australian Synchrotron XAS beamline using the hybrid technique (Chantler *et al.*, 2012*b* ▸, 2015 ▸; Best & Chantler, 2022 ▸) to obtain XAFS data for zinc metal foils at temperatures of 10 K, 50 K, 100 K and 150 K. Zinc foils with nominal thickness of 10 µm and 50 µm were sourced from Goodfellow with reported purities of 99.9% and 99.95%, respectively. The foils were rolled, which can provide some static disorder from twinning or stacking faults, compared with an ideal crystal structure for example, but the foils exhibited no obvious pattern or defects. Mounts had low stress so had no impact on the metal nanostructure. They were placed inside a cryostat at 45° from the incident beam with a 100-pixel monolithic germanium detector, the fluorescence detector, placed at 90° (Fig. 1 ▸). Three ion chambers were utilized in the setup with one upstream (*I*_0_), one downstream (*I*_1_) and a third used to collect reference foil information with reference foils situated between *I*_1_ and *I*_2_ for energy calibration. A daisy wheel with different aperture sizes and range of aluminium foils was placed between the sample and each of the first two ion chambers.

**Figure 2:**
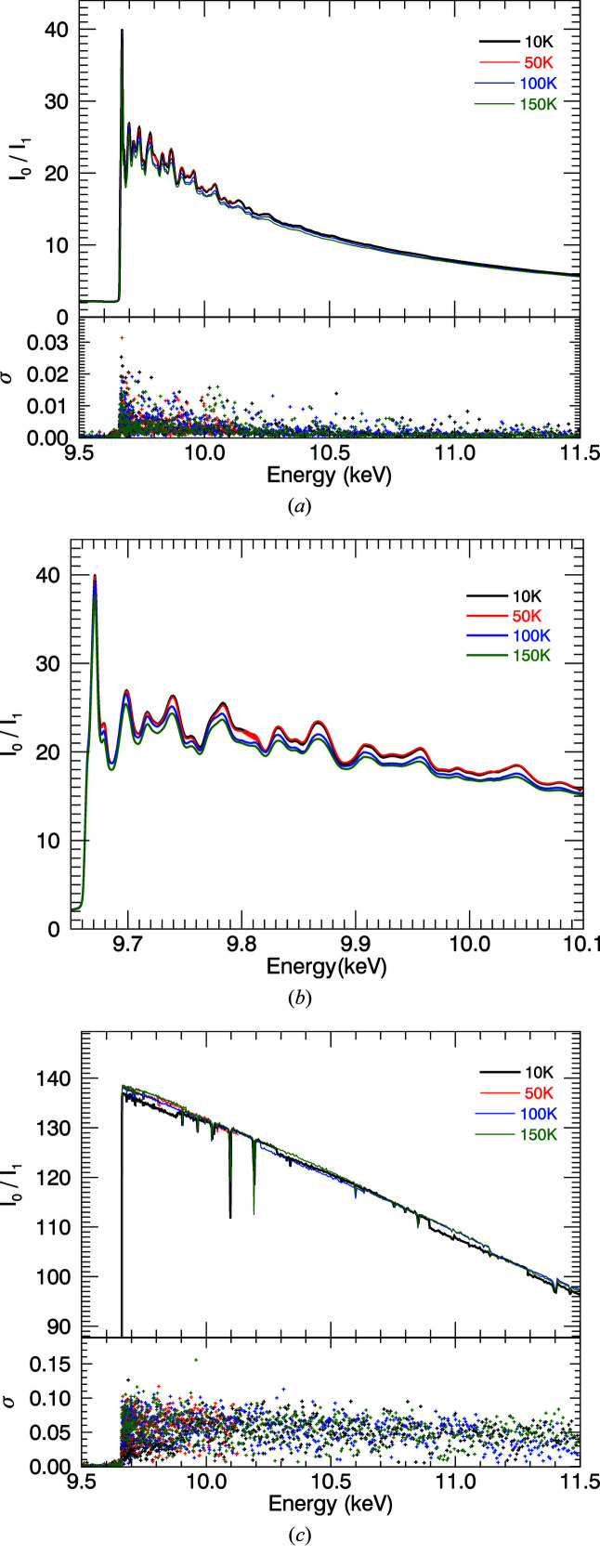
Transmission ratio of the downstream (*I*_1_) ion chamber normalized by the upstream (*I*_0_) ion chamber for (*a*, *b*) the 10 µm foil and (*c*) the 50 µm foil. The 10 µm data show highly detailed XAFS well beyond the edge, and temperature variation. The σ reported in (*a*) and (*c*) is the standard error of three repeated measurements, <0.1% for the 10 µm foil. All XAFS is lost in the 50 µm data, where *I*_1_/*I*_0_ ≃ exp(−10).

**Figure 3:**
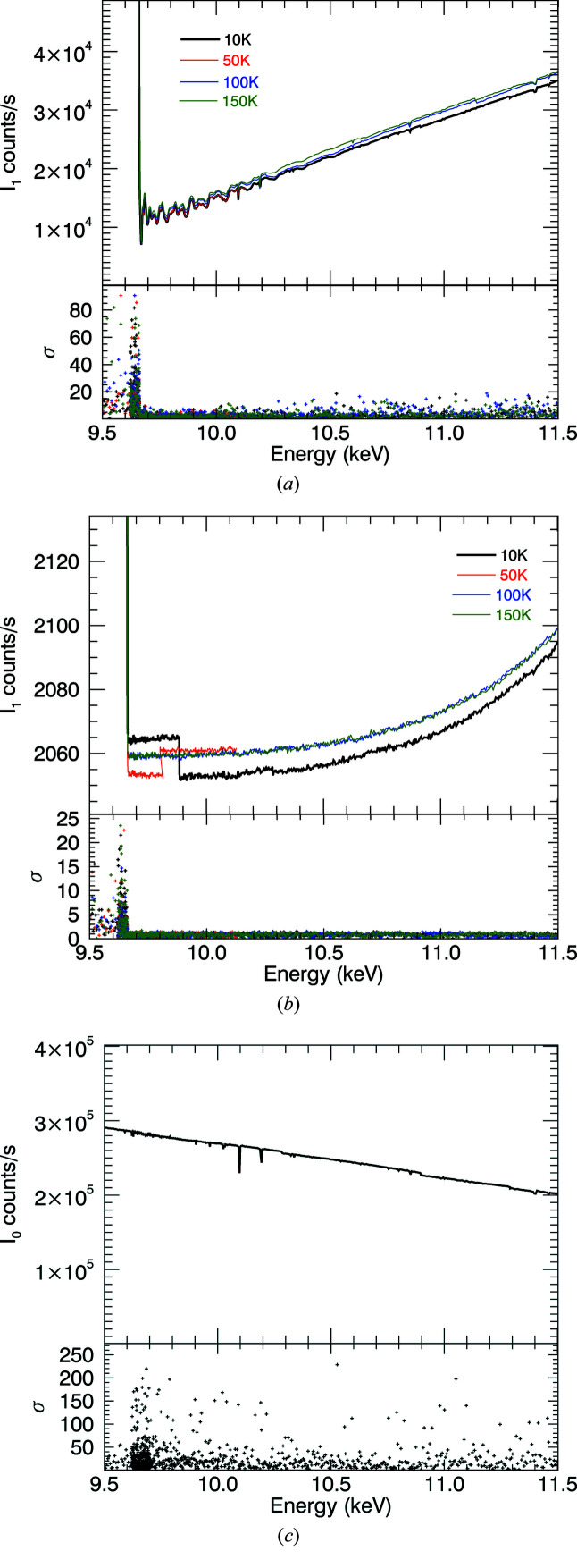
Raw counts and experimental standard errors σ of three repeated measurements of the upstream and downstream ion chambers. Temperatures are given for the (*a*) 10 µm and (*b*) 50 µm foils. The 50 µm foil attenuated excessively towards the dark-current limit. Pre-edge counts read 1.3 × 10^5^ and 3.5 × 10^4^ for the 10 µm and 50 µm foils, respectively. (*c*) *I*_0_ is independent of temperature and remained relatively consistent with the exception of Bragg glitches.

Fig. 2 ▸ presents the ratio of upstream (*I*_0_) and downstream (*I*_1_) ion chamber readings. Each point of the spectrum was measured three times providing a point-wise statistical uncertainty for each ion chamber reading. The high quality and resolution of the spectra are immediately visible by the detailed oscillations, low uncertainty and height of the first peak relative to the background. Fig. 3 ▸ reports the counting rates and statistics for the individual ion chambers. Foil thicknesses are chosen according to the expanded Nordfor’s criterion (Nordfors, 1960 ▸; Chantler *et al.*, 2000*a* ▸,*b* ▸, 2012*a* ▸) with electronic settings optimized for counting statistics. At the highest level of absorption, the 10 µm foil maintains highly detailed structure and statistics, whilst the 50 µm foil goes below the noise floor.

The upstream ion chamber shows a very smooth response function, with some Bragg peaks (Bragg glitches) that are characteristic of the Si(111) double-crystal monochromator used to monochromate the desired photon energy. Due to the high linearity of the ion chambers, these Bragg peaks dis­appear when normalized with the upstream counts. Fig. 2 ▸ shows the result of taking the ion chamber ratio for each temperature. Increased broadening for higher temperatures is already visible.

### 2.1. Dark current characterization

The current amplifier provides the option to null the offset voltage; but doing so makes one blind to the actual value and its variation if negative, and destroys the detector linearity. So, we set the current offset so that the values are always within range; in principle, this does not compromise the sensitivity of the measurement. The resulting non-zero values are referred to as a dark current, the current measured in the absence of X-rays.

**Figure 4:**
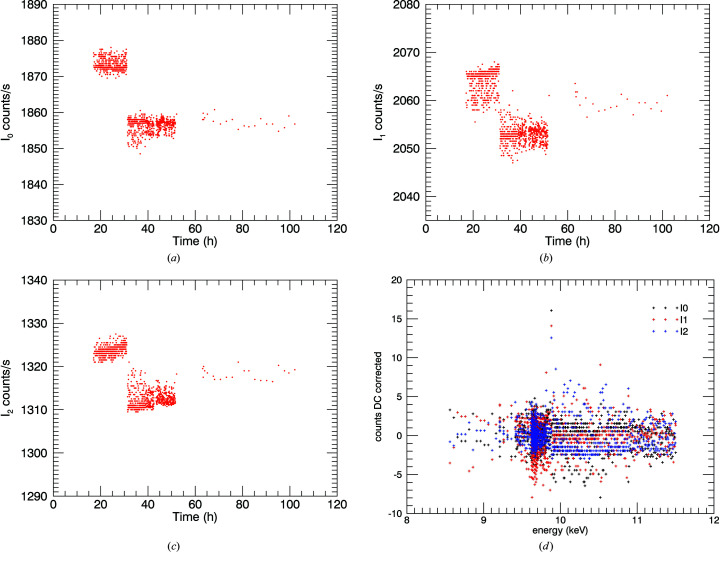
Dark-current measurements of each ion chamber (*a*) *I*_0_, (*b*) *I*_1_, (*c*) *I*_2_. The dark current can then be subtracted. The discontinuity follows a change in detector gain. (*d*) This noise is included in the variance in counts in the uncertainty of the EXAFS spectra.

This was measured throughout the experiment by taking readings at regular intervals with a physical shutter blocking off all incident X-rays (Fig. 4 ▸). The counts from the X-ray source are isolated by subtracting the dark-current counts from the total in each of the ion chambers. The intensity ratio due to the change in X-ray flux hence then becomes 

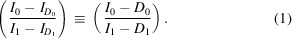

**Figure 5:**
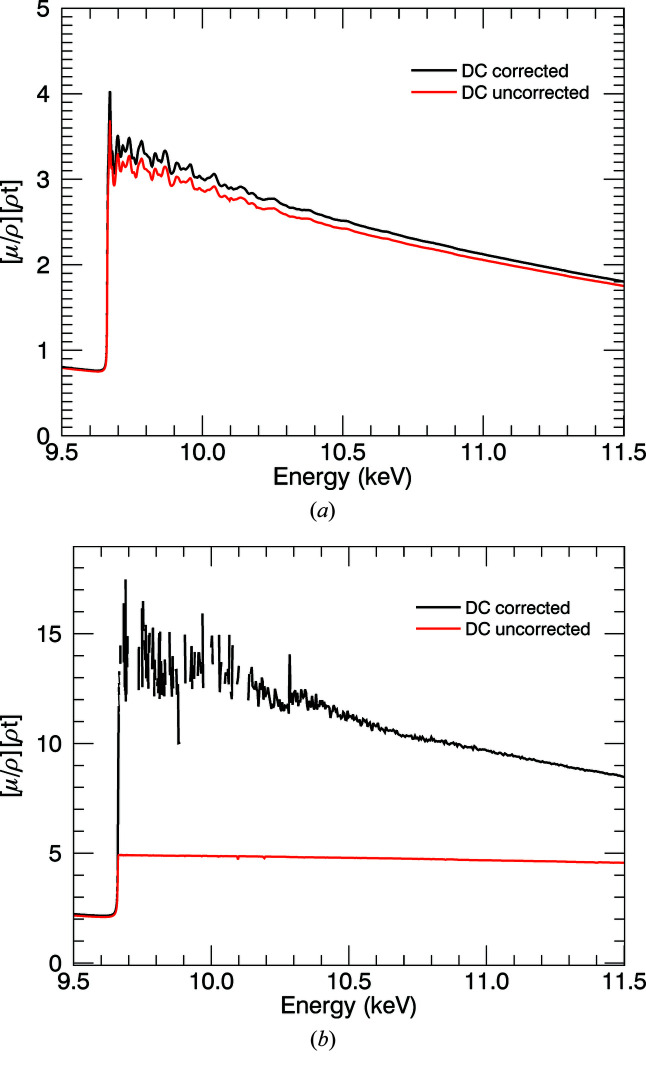
The effect of correcting for dark current in the 10 K data for the (*a*) 10 µm foil and (*b*) 50 µm foil. At 10 µm, the largest displacement is near the absorption edge. The largest glitch in the uncorrected data, near to 10.1 keV, disappears after correction for dark current. For 50 µm, correcting for the dark current reveals some consistency in the spectra at high *k*. It is quite important to monitor the dark current through a spectral data collection.

There are three distinct regions, corresponding to different amplifier tunings and to changing coupled background electronic noise from the beamline. An average or linear model [*A* + *B*(*t* − *t*_0_)] is fitted in each region. We observe any changes of the dark current by monitoring it through the experiment. The effect of this correction is largest just above the edge, where the dark current makes a larger contribution to downstream ion chamber readings. Here, it increases ln(*I*_u_/*I*_d_) by 8.7% for the 10 µm foil, while away from the edge at 11 keV this is around 3%, which continues to be significant. The change in the spectra for the 50 µm foil (Fig. 5 ▸) reveals that useful information can be extracted at a high energy, and reveals the power of accurate characterization of the dark current.

The standard error of the normalized signal with dark-current correction [Fig. 4 ▸(*d*)] is 

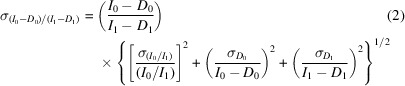

where *I* represents the counts in each ion chamber and *D* is the corresponding dark-current count; ‘0’ refers to the upstream detector, ‘1’ refers to the downstream detector. Each σ is calculated as a standard error. In Fig. 5 ▸ the glitch in the uncorrected data at 10.1 keV lines up with the multiple Bragg reflection in Fig. 3 ▸(*c*). This disappears after dark-current correction, as the detectors then have a higher linearity. The numerical corrections applied are summarized in Table 1 ▸. It is clear that: (1) dark-current monitoring and correction can be successfully accomplished with a cryostat in low-temperature studies in transmission; and (2) the corrections are very important for thin and thick samples.

### 2.2. Blank measurements and correction for the beam optic

The beam is attenuated by everything in its path – windows into and out of the ion chambers, the cryostat, and air path attenuation. The total attenuation is

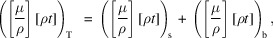

where subscript s represents attenuation due to the sample and b (blank) represents attenuation by everything else in the beam path between the two ion chambers. We take measurements without any sample to find the absorption of the integrated path. This blank measurement was completed at each energy, so the correction can be applied and experimental errors propagated in a point-wise manner, 

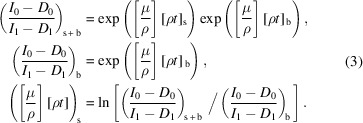

The uncertainty is then (Chantler *et al.*, 2012*a* ▸)

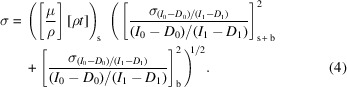

**Figure 6:**
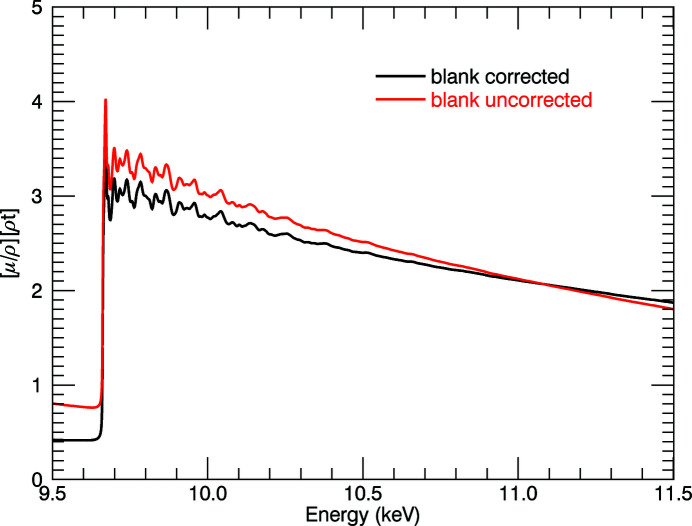
Change in spectra due to blank correction at 10 K for the 10 µm foil. Red: corrected for dark current; black: corrected for blank and dark current, with a reduction of 52.9% pre-edge, and significant change to the slope.

The ‘blank measurements’ are energy dependent and depend on the relative gain of the ion chambers. In Fig. 6 ▸, at the edge, the systematic effect is in the negative direction, which is opposite to that of the dark current. Removing this effect from the data reduces the attenuation coefficient by up to 52.9% in the pre-edge region, while at higher energies it is increased by up to 3.84%. At the attenuation peak these effects are of similar magnitude, but the combination makes a change to the shape of the distribution.

**Figure 7:**
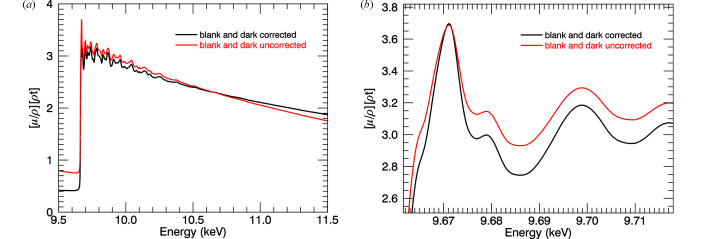
Changes to the 10 K absorption spectra, for the 10 µm foil, due to dark current and blank corrections. (*a*) Changes to the shape of the slope, pre-edge and edge-jump. (*b*) The near-edge structure shows significant change in form for processed data with sharper peaks.

Correction for dark current and blanks contribute to the atomic background of mass attenuation spectra and also to the XAFS oscillations, significantly increasing their sharpness. Changes to the XANES structure and to the *k* < 3 XAFS are very important (Fig. 7 ▸). Hence any theory to understand the XANES, early XAFS or pre-edge should include blank and dark-current monitoring and correction.

**Figure 8:**
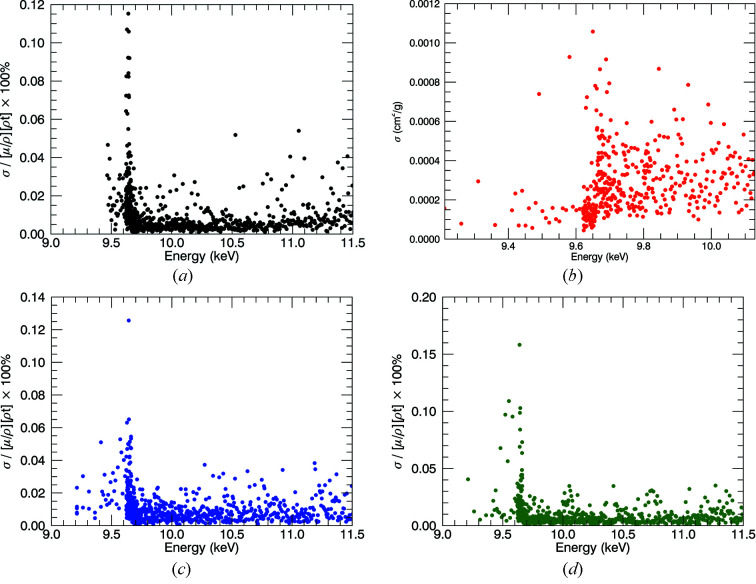
Uncertainties 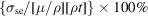 for (*a*) 10 K, (*b*) 50 K, (*c*) 100 K and (*d*) 150 K. Typical uncertainties are below 0.1% at the edge and below 0.03% thereafter, in line with earlier work (Sier *et al.*, 2020 ▸; Ekanayake *et al.*, , 2021*a* ▸,*b* ▸).

Experimental uncertainties of 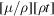 using error propagation in equations (2) and (4) are shown in Figs. 8 ▸(*a*)–8(*d*). The typical accuracy of all measurements to this point are below 0.03%, rising to 0.1% at the absorption edge. It is clear that: (1) dark current and blank monitoring and correction can be successfully accomplished with a cryostat in low-temperature studies in transmission; and (2) the corrections are very important for thin and thick samples.

## 3. Additional systematic corrections and the hybrid technique

**Figure 9:**
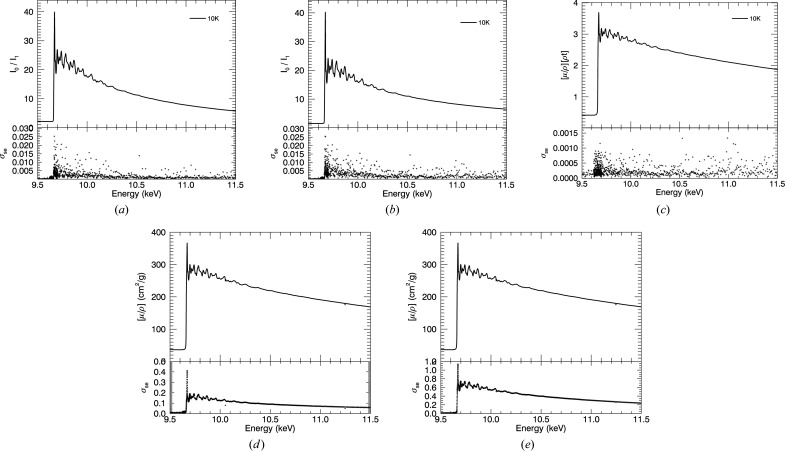
Summary of the progression of systematic corrections and the associated standard error after each correction, for 10 K. (*a*) Ratio of raw counts of upstream and downstream ion chambers with standard error derived from repeated measurements. (*b*) Ratio of ion chambers after correction for dark current. (*c*) [μ/ρ][ρ*t*] from taking the logarithm of (*b*). (*d*) Converting to units of the mass attenuation coefficients and correcting for scattering, according to equation (14). (*e*) Corrected measurements with roughness correction and corresponding uncertainty.

The monitoring of dark current and blank measurement can lead to large structural corrections to the data (Fig. 9 ▸). The measurements of sample, blank and dark current are intrinsic to both XERT and hybrid techniques. Other systematics that can be very significant are detailed in Appendix *A* [App appa] [App appb] [App appc] [App appd] [App appe], including: harmonic content of the beam on the sample; energy calibration; thickness and integrated column density determination, and hence the absolute value for the attenuation coefficient; scattering and fluorescence, which yielded a significant correction; roughness of the samples; and bandwidth of the synchrotron beam on the sample. Because the sample was small and could not be mapped, the absolute value of thickness, integrated column density and hence 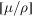 could not be directly measured. However, the nominal or supplier thickness is often quite inadequate. Hence the hybrid technique here uses an independent reference measurement where XERT has been applied and, at least at 298 K, can be directly transferred to normalize and calibrate the absolute value in transmission. Similarly, XERT should measure the energy of the beam at the sample independently and directly. However it is important to realize that a direct measurement of beam energy should be made to enable absolute measurements to be made. But the beamline had no independent measure of energy, so we used, with some limitations of accuracy, selected reference foils with some provenance and the internal edge measurement from the data to calibrate the energy. The measurement of systematic errors requires multiple samples (thicknesses or concentrations) in XERT to calibrate harmonics, fluorescence, roughness and bandwidth; however, this experiment had only two samples and effectively one high-accuracy sample; so estimates and characterization of this, using the hybrid technique, followed a separate XERT measurement on a reference sample. These were all characterized fairly well, with small uncertainty, but indirectly and with some increase in uncertainty as stated. One advantage of the hybrid technique presented here is that it gives a direct insight into systematics and magnitudes for the corresponding, simultaneous fluorescence measurement, discussed later. This is also part of the hybrid technique – to investigate and characterize fluorescence spectra.

### 3.1. Transmission spectra and uncertainty contributions

**Figure 10:**
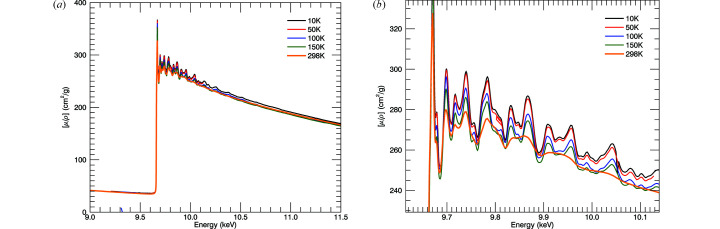
(*a*) Full temperature series of mass attenuation coefficients including room-temperature data from the previous XERT experiment. (*b*) Low-*k* oscillations.

Fig. 10 ▸ provides the X-ray mass attenuation coefficients for the temperature series including room-temperature data. We were able to investigate or measure several important systematics which can strongly affect many different parameters of the experiment. These include; the energy *E* or *k* axis, the amplitude, attenuation or photoabsorption 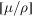 axis, and the structure of the XANES and XAFS oscillations, including dark-current monitoring, blank monitoring, harmonic, thickness calibration, fluorescence, roughness and bandwidth. Importantly, we investigated these for measurements in a cryostat in transmission even though we cannot in this case use a full XERT approach and therefore are using a hybrid approach. There is a large significant difference between the 150 K and 298 K room-temperature spectra, as hoped. All low-temperature spectra show additional resolved peaks at 9.86 keV and 9.94 keV. For 10 K, 100 K and 150 K, the data extend above the zinc *K*-edge to 11.5 keV; for 50 K, the data extend to 10.1 keV. The error contributions in the absorption spectra are summarized in Table 2 ▸. The key contributors to the uncertainty are the counting statistics and the uncertainty in foil thickness. Although the numerous contributions we have discussed produce significant systematic effects on the mass attenuation coefficients, they are well characterized and so after correction they contribute very little to the total uncertainty. The typical uncertainty in the mass attenuation coefficients is 0.033%.

## 4. Evolution of nanostructure

### 4.1. Background subtraction

The XAFS can be isolated from the background,

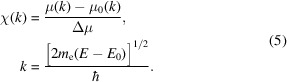

μ_0_ is the smooth atomic background; Δμ is the change in absorption from before the edge to the peak of the atomic background, also known as the edge jump; *E*_0_ is the energy of the absorption edge; *m*_e_ is the mass of the electron; and ℏ is Planck’s constant.

**Figure 11:**
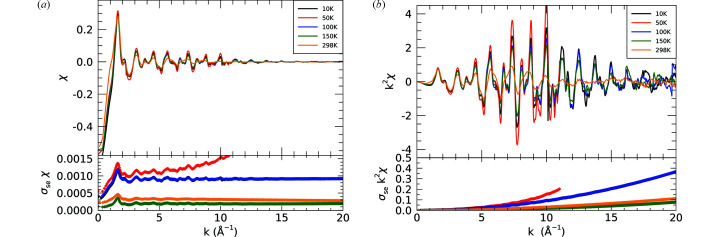
(*a*) Temperature series of χ(*k*). The 50 K data set was collected with a much narrower *k*-range, up to *k* = 11 Å^−1^. (*b*) Temperature series of *k* ^2^χ(*k*) showing the structural consistency and yet the change in sharpness with temperature, with resolved peaks to *k* of 17 Å^−1^ for low temperatures.

The software *mu2chi* (Schalken & Chantler, 2018 ▸) follows the common method of fitting a segmented cubic spline; however, it has the advantage of avoiding interpolation of the data onto a uniform *k*-spaced grid. Hence no information is lost and it also propagates the experimentally derived point-wise uncertainties to allow a properly weighted fit. Fig. 11 ▸ demonstrates well resolved sharp peaks in χ being magnified by over an order of magnitude for the 10 K compared with the 298 K data. The low-temperature data resolves peaks all the way to *k* ≃ 17 Å^−1^ compared with 13.5 Å^−1^ at room temperature. The broadening at room temperature obscures peaks at *k* = 7, 9.5 and 11.5 Å^−1^.

### 4.2. Nanostructure

Using μ_TOTAL_ = μ_0_(1 + χ),

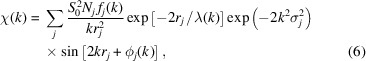

we use *eFEFFit* (Smale *et al.*, 2006 ▸; Schalken & Chantler, 2018 ▸), which takes the contributions of each potential scattering path theoretically calculated by *FEFF* and fits them to the XAFS equation, following the popular *iFEFFit* but with propagated uncertainty. Key parameters are: coordination number (*N*_*j*_); amplitude reduction factor ( 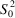 ); disorder captured in the Debye–Waller factor ( 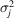 ); and effective path distance (*r*_*j*_), which can be parameterized by a scaling factor α_*j*_.

The coordination number and inner shell path distances are defined by zinc hexagonal close packing (Ledbetter, 1977 ▸). Beyond the nearest dominant coordination shells, a single correction factor α can be sufficient. 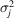 is estimated from educated guesses about the relations between paths, to reduce the total number of fitting parameters. These follow guidelines by Ravel (2016 ▸) and Hudson *et al.* (1995 ▸) and involve trialling different groupings of path parameters in the XAFS equation to minimize 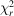 . The transition structure changes at each temperature in the temperature series, especially due to anisotropic thermal expansion and, in other materials, phase changes.

**Figure 12:**
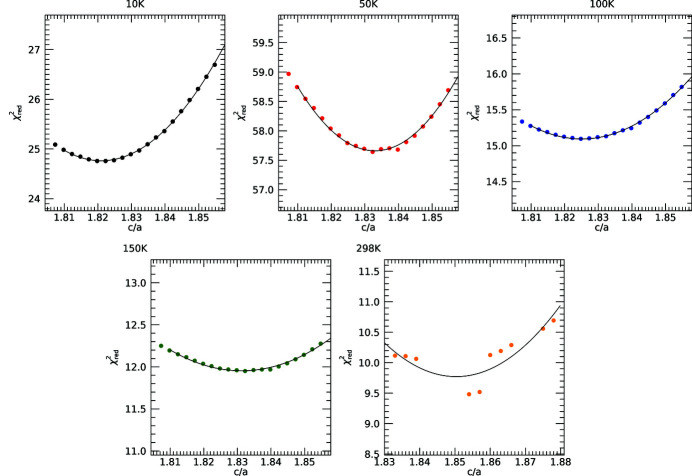
Results of a grid search of *c*/*a* in steps of 0.003 with *eFEFFit* yielding smooth quadratics (Table 3 ▸).

Nuss *et al.* (2010 ▸) measured the change in lattice parameters with temperature using X-ray diffraction and found that the expansion was not isotropic; in the hexagonal plane it did not contract below 150 K. Since *a* is stable at low temperatures (Nuss *et al.*, 2010 ▸), we investigated varying the out-of-plane axis *c*. We implement a grid search through *c*/*a* (out-of-plane/in-plane ratio) (Fig. 12 ▸). This provides a measure for the lattice spacing and ratio coinciding with a minimum of the 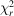 best-fit parameter.

**Figure 13:**
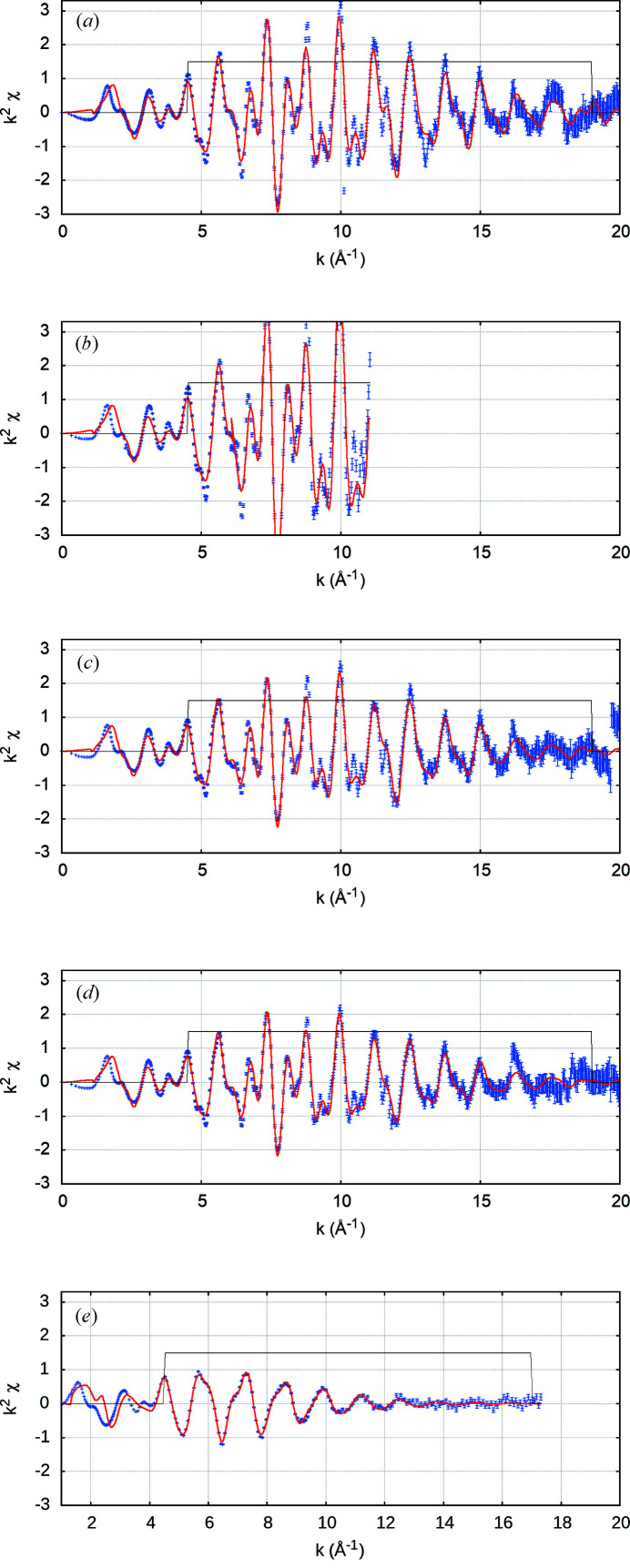
Results of *eFEFFit* modelling at (*a*) 10 K, (*b*) 50 K, (*c*) 100 K, (*d*) 150 K, (*e*) 298 K using initial geometry from Nuss *et al.* (2010 ▸). The *FEFF* fits match the structure very well across a large *k*-range and also in the lower *k*-range. At increasing temperature, the oscillations are of smaller magnitude and less detailed.

A model based on the 10 K data set analysis was used to provide initial estimates to higher-temperature data sets to minimize the correlation of parameters. A *k*-range of 4.5–19 Å^−1^ was used as optimal. Fitting below this range led to a significant increase in 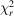 and introduces significant discrepancies with oscillations at higher *k* – that is, the theory was discrepant below this range. Above 19 Å^−1^, or 17 Å^−1^ for 298 K, the uncertainties dominated over the oscillations. The shortest 37 independent scattering paths (two-leg and three-leg) were modelled. Five scaling and thermal correlation parameters were used in the fit, one for each of the first three independent shortest single leg paths, another to the next 19 longer (*r*_eff_ < 5.32 Å) and one for the remaining 15 (5.32 Å < *r*_eff_ < 7 Å) (Fig. 13 ▸ and Table 3 ▸). The many-body reduction factor should be smaller than 1.0, yet, if left to fit freely, correlations lead to it exceeding 1.15. Fixing 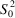 = 0.9 following Ekanayake *et al.* (2021*a* ▸) was tested, but did not result in any significant change in *c*/*a* or fitting parameters.

Each increasing temperature has a smaller magnitude of oscillations and less detailed structure, which reduces 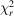 (Fig. 13 ▸). At lower temperatures, some fitted peaks are not as resolved as the experiment, and some of the sharper peaks are not resolved which can be due to fitted σ_*j*_ being too large. Parameter uncertainties are similar. The increasing sharpness of structures at low temperatures yields deviations of greater significance from the fit, while the structures and fits are qualitatively similar.

## 5. Fluorescence: experimental

**Figure 14:**
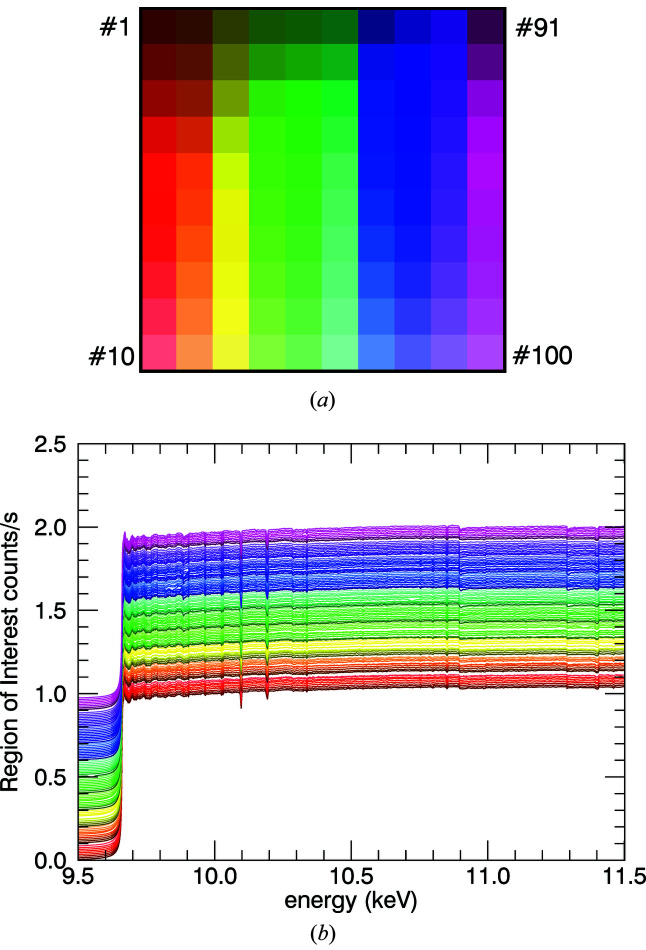
(*a*) Colour palette for each pixel in fluorescence plots used in this paper. Some plots appear dominated by pink/purple colours because higher-numbered pixels are plotted last. (*b*) ROI normalized by edge height and offset according to pixel number. Little divergence between pixels is seen.

Fluorescence data were taken using a 100-pixel monolithic germanium detector with a total active area of 5 cm × 5 cm and depth 7 cm. The beamline software and detector recorded three channels of data: an input count rate (ICR, fast), output count rate (OCR, slow) and integrated region of interest (ROI) (Fig. 14 ▸). The third channel only counts photons within the desired energy range around 8.627 keV which is the energy of Zn *K*α. The other two channels are used to correct for detector non-linearities. In fluorescence mode, the relative attenuation coefficient does not correct for systematic effects, 

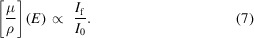




### 5.1. Pixel dead-time corrections

Pixel dead-times are quoted as 0.83 µs and 4.73 µs for input and output count rates, respectively. This XAFS facility does not record the full spectra from the fluorescence detector, in part because of data transfer times and binary storage space. Input and output count rates (ICR and OCR, respectively) can be used to correct the integrated ROI counts,

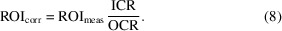

Non-linearity in pixel response will result in dampening of the XAFS spectrum. Compared with the uncorrected data for a typical pixel, the magnitude is increased by over 20%. Unlike in well characterized absorption data, with matching detectors with high linearity corrected for dark and blank readings *etc*., the different structure and properties of the fluorescence detector, and limited linearity ranges, means the Bragg peaks (glitches) in intensity do not normalize and cancel out; hence these individual or sometimes two neighbouring data points will have to be deleted during further processing.

## 6. Advances in self-absorption correction

**Figure 15:**
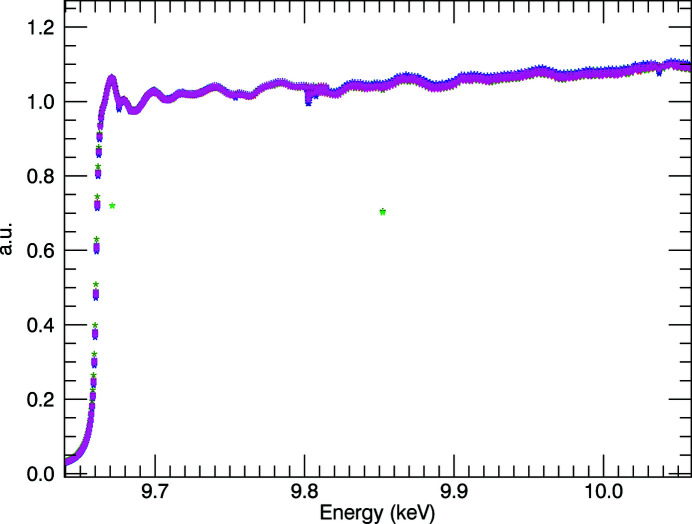
150 K fluorescence data with each pixel corrected for dead-time, normalized by *I*_0_ and edge height, plotted following Fig. 14 ▸. The characteristic rising trend almost always seen in fluorescence spectroscopy is clear and a characteristic signature of absorption of the incident beam, in the sample.

There are two major sources of systematic error when performing XAS experiments in fluorescence mode. The spectra have a rising gradient (Fig. 15 ▸) instead of a negative gradient which would correspond with absorption coefficients in transmission, or the measurement of absorption. For multi-pixel detectors, a divergence in the pixels occurs due to the dependence of self-absorption in the sample on the detector geometry. As a photon penetrates the sample, it is absorbed at some depth, and the lower-energy fluorescence photon is absorbed again by the sample before making it to the detector. Photons that penetrate deeper then have a higher chance of the fluorescence photon being absorbed within the sample, which creates a gradient across the detector. At low attenuation coefficients, *e.g.* as one goes to higher energies, the photons penetrate deeper into the sample, and a divergence in multipixel spectra is often observed (Trevorah *et al.*, 2019 ▸). An example for the ratio of the dead-time-corrected fluorescence signal to the incident count is given in Fig. 15 ▸.

Other experimental conditions – such as the use of thin foils, grazing or low angle of incidence, near-90° emission angle, and distance between sample and detector – can help to mitigate these pixel-dependent effects but inevitably the count rate is reduced. To correct for these effects (Chantler *et al.*, 2012*b* ▸), the program *SeAFFluX* (Trevorah *et al.*, 2019 ▸) was adapted to suit the relevant experimental geometry and the 100-element Ge detector.

The code takes in an experimental geometry and uses *FFAST* theoretical calculations to fit the self-absorption functional equation (9),

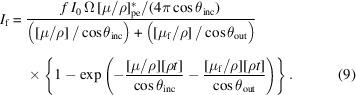

**Figure 16:**
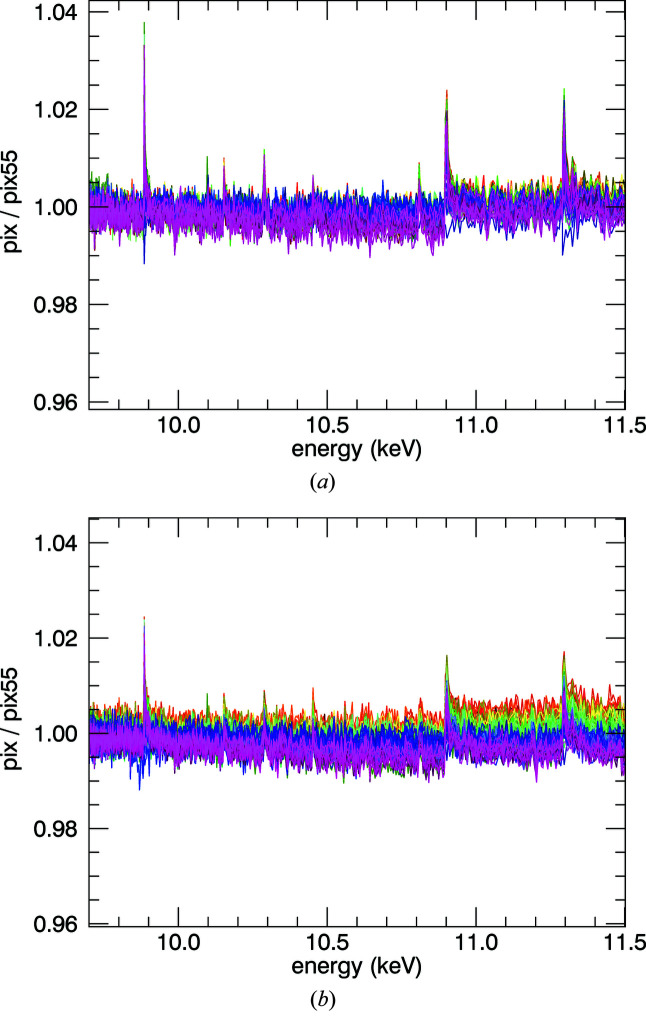
The spread of pixel intensities for the (*a*) 10 µm foil and (*b*) 50 µm foil across the whole above-edge energy range. Fluorescence intensities in each of the 100 detector elements, normalized by the upstream ion chamber counts at the absorption edge (9.671 keV), normalized to pixel #55 (close to the centre), across a broad range of energies. For the 10 µm foil, the spread is due to the low count rate and noise, *i.e.* the experimental uncertainty. For the 50 µm foil, there is a very clear divergence between pixels, caused by self-absorption. The intensity decreases as we move across the detector, as predicted by self-absorption (Fig. 14 ▸). *SeAFFlux* provides geometrical, self-absorption and mode (fluorescence, attenuation) corrections to convert fluorescence intensity into a quantitative 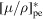 , ready for analysis using traditional XAFS packages. *SeAFFluX* uses the experimental geometry to model the divergence and correct accordingly for each pixel, rather than just averaging the pixels.

Here, θ_inc_ and θ_out_ are the angles of the incident X-rays and outgoing fluorescence, respectively. [μ_f_/ρ] is the mass attenuation coefficient of the material at the energy of the fluorescence photon. The penetration depth into the material is more relevant than the material thickness. *f* is the fluorescence yield, the fraction of events leading to a fluorescent photon emission. Fig. 16 ▸ suggests that conditions avoided major divergence of pixel spectra seen in previous fluorescence experiments (Trevorah *et al.*, 2019 ▸) – that is, the fluorescence detector was a significant longer distance from the cryostat. The *SeAFFluX* code, adapted for this experimental setup, has corrected the gradient of the spectra very well.

**Figure 17:**
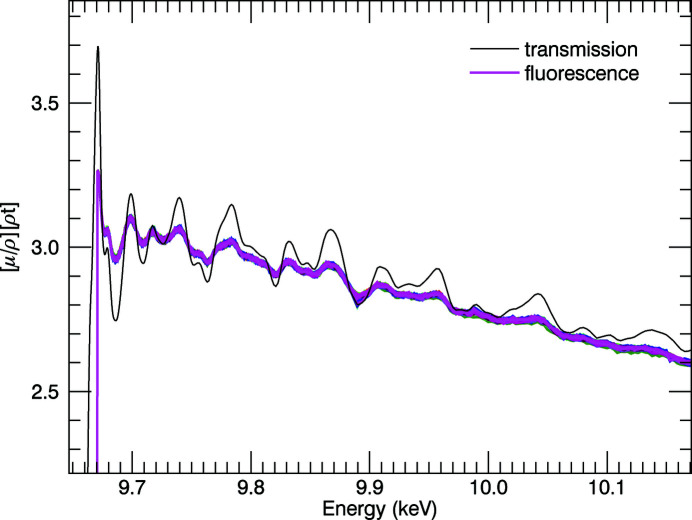
Near-edge region comparing the transmission spectra with data collected in fluorescence mode and processed by *SeAFFluX*. The form matches very closely but the magnitude of the XAFS in fluorescence is ∼0.3 that of transmission.

**Figure 18:**
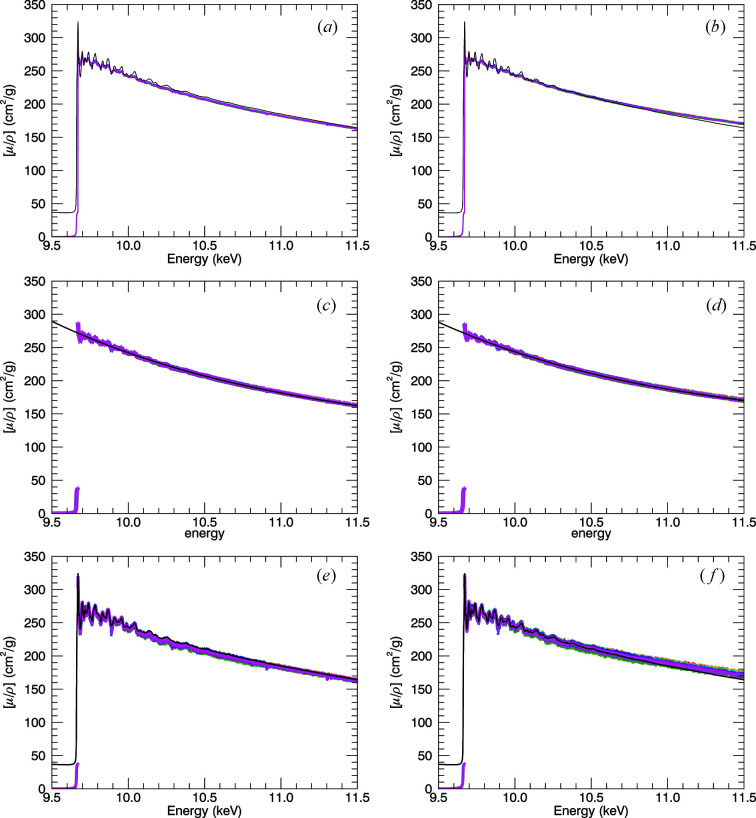
Processing incorporated into *SeAFFluX* to amplify oscillations and return an accurate set of mass attenuation coefficients. The left hand column (*a*,*c*,*e*) is 10 µm foil data and the right hand (*b*,*d*,*f*) is 50 µm foil data from the 10 K spectra. Panels (*a*) and (*b*) demonstrate the output of *SeAFFluX* and the shape of the slope. The 10 µm foil transmission spectrum is plotted (black line) in all panels. Panels (*c*) and (*d*) show basic spline fits; (*e*) and (*f*) show results after scaling the divergence relative to this spline.

The obvious discrepancy of the two sets is that the magnitude of the XAFS oscillation differs by a factor of ∼3 (Fig. 17 ▸). Fluorescence-detector ROIs exclude photons below the desired edge, so that the baseline scaling of the structure can be misaligned and hence mis-scaled. Equation (9) is highly affected by strong oscillations compared with a background or spline scaling, so for example a strong peak (here, ln ratio ≃ 3.7) is much more heavily damped and a strong valley (here, ln ratio ≃ 2.7) is (much) less damped. This variation depends upon the emission angle, the log ratio of attenuation and therefore particularly the oscillation magnitude. Here, *SeAFFluX* uses reference atomic theory, which translates the baseline very well but does not correspondingly scale the peaks and troughs which have strong differential scaling amplification relative to the baseline. So while the base and spline background level is well corrected here (ln ratio ≃ 3 towards ≃ 2 above the edge), the data and amplitudes should be used for the amplification of the oscillations – but in self-absorption these are heavily damped so that the signatures for this in the data are weak. To compensate for the low-magnitude XAFS, a cubic function was fitted through the spectrum and deviations from it are amplified to match the magnitude of transmission XAFS (Fig. 18 ▸). As the oscillations are amplified, the noise is correspondingly amplified.

### 6.1. Fluorescence spectra and uncertainties

**Figure 19:**
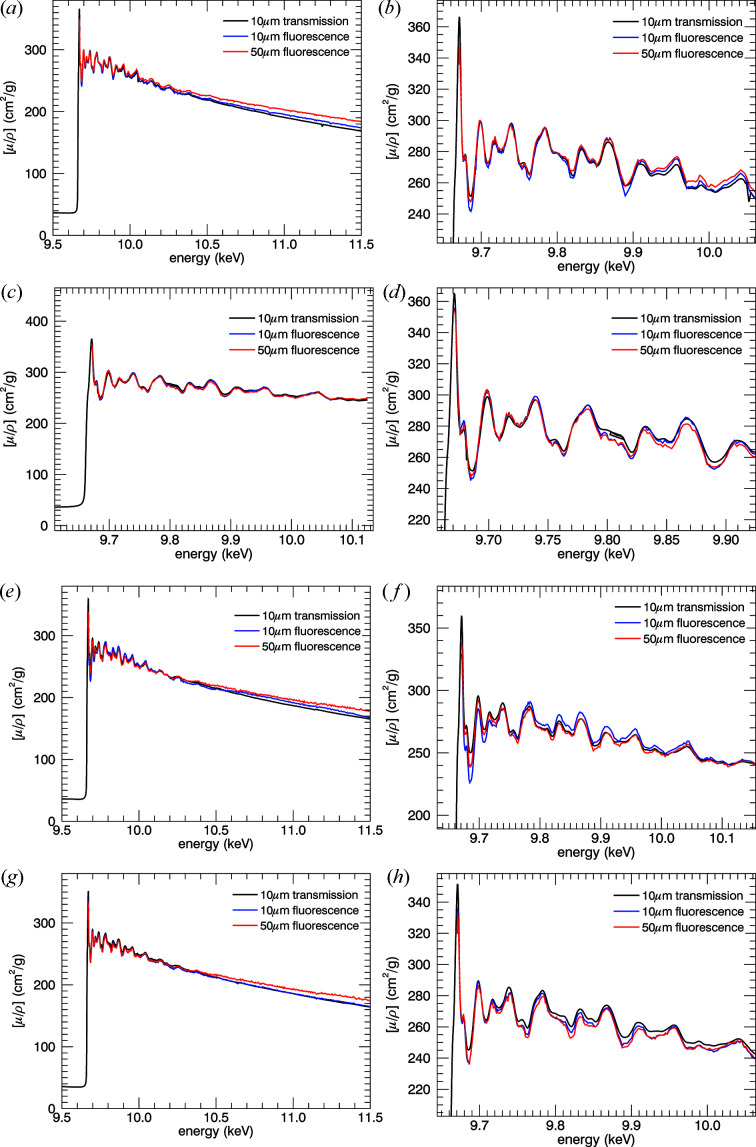
Results from modified *SeAFFluX* after rescaling and averaging over pixels. From top to bottom: 10 K, 50 K, 100 K, 150 K. The fine structure of the XAFS (right) matches perfectly with small overarching deviations due to the spline fitting. The noise is visibly larger in the fluorescence spectra than in transmission, but all the detail of the XAFS is clearly visible. With careful analysis, just as much structure can be seen in fluorescence analysis as in transmission.

**Figure 21:**
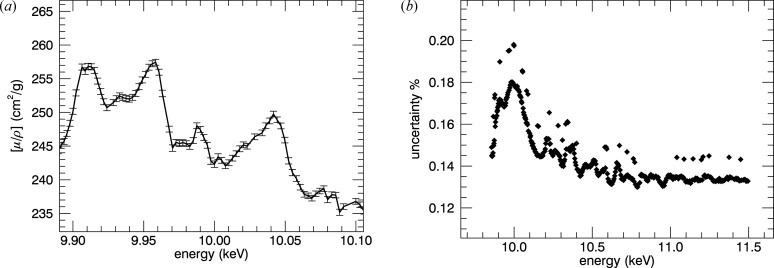
(*a*) A sample of the spectra with uncertainties from the 10 K, 50 µm fluorescence spectra. (*b*) The percentage uncertainty in the fluorescence spectra of 10 K, 50 µm.

Figs. 19 ▸ and 20 ▸ demonstrate results from processing the fluorescence data which produce a very high level of information content, comparable with that in transmission. The typical uncertainty in the fluorescence spectra in 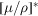 is between 0.13% and 0.18%, comparing favourably with the transmission data [see Figs. 11 ▸ and 9 ▸ from (0.6–1.0)/300 → 0.25/170 ≃ (0.2–0.3)% and hence → 0.15% in 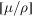 ; and correspondingly 0.0003–0.001–0.0015 in χ]. The uncertainties are generated by repeated measurements and point-wise propagation by adding uncertainties of dead-time measurements in quadrature. The uncertainties match the level of noise (Fig. 21 ▸), confirming that the experimental uncertainties are accurate, to within a factor of two.

### 6.2. Evolution of nanostructure from fluorescence data sets

**Figure 22:**
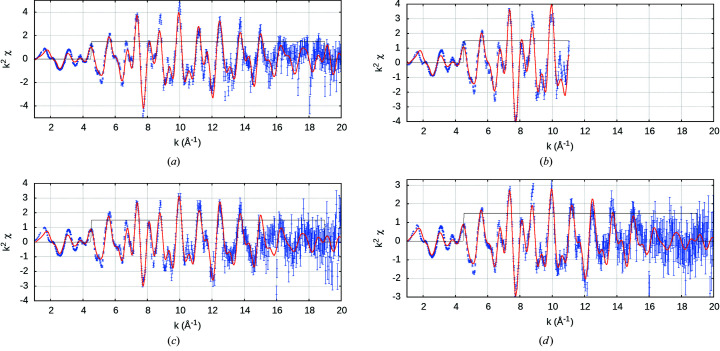
Fluorescence XAFS spectra in *k* ^2^χ versus *k* and theoretical fits at (*a*) 10 K, (*b*) 50 K, (*c*) 100 K and (*d*) 150 K. The fit follows the data very closely, even below the fitting window.

We use *mu2chi* to transform the corrected normalized fluorescence spectra in 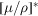 versus *E* into the XAFS χ(*k*). However, the normalization of the oscillations is dependent upon the edge jump Δμ and the ln ratio in equation (5). Fluorescence measurements are unreliable (zero) pre-edge due to the ROI, so there is no defined edge jump. We include the pre-edge region of the transmission data set to give the appropriate edge jump ratio. Since the fitting procedure only applies above the edge, this provides an accurate amplitude for χ (Fig. 22 ▸). Theoretical fits are then in close agreement with experiment. A strong endorsement for the physical relevance of the data and fit is the continued agreement in the *k*-range below the Hanning window at 4.5 Å^−1^, even down to 2 Å^−1^.

The model for each spectrum is identical to that in transmission. 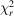 is significantly lower for fluorescence, 4.75 < 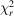 < 15.6 (Table 4 ▸), compared with Table 3 ▸ for transmission, of 11.9 < 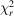 < 57. *Per se*, this is not an argument for neither data set nor fit. Most α_*j*_ and σ_*j*_ values are consistent within the uncertainties in both transmission and fluorescence. α_1,4_, σ_1,4_ correspond to single-scattering paths entirely within the hexagonal plane and α_2,3_, σ_2,3_ have an out-of-plane component. α_*j*_ and σ_*j*_ increase with path length and the σ_*j*_ increases with temperature. The asymmetry in σ_*j*_ between in-plane and out-of-plane scattering paths is corroborated by Rae *et al.* (2010 ▸). The key difference between transmission and fluorescence is 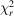 , which commends both spectroscopies.

## 7. Evolution of *c*/*a* from transmission and fluorescence datasets

**Figure 23:**
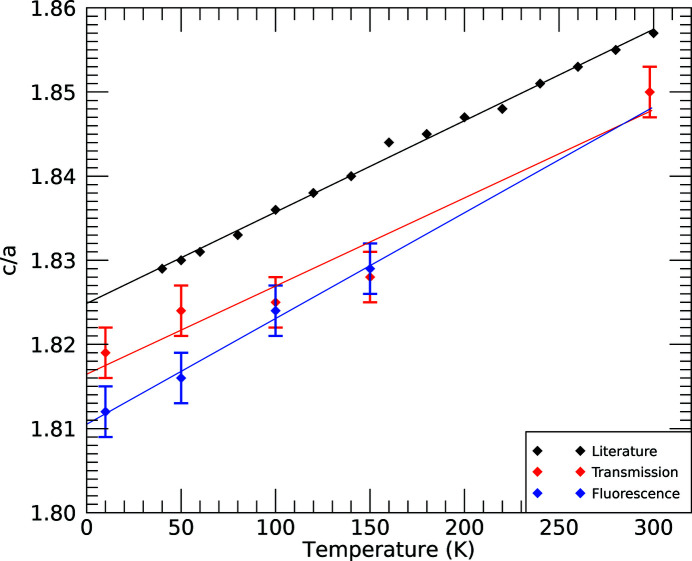
Optimized 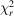 for *c*/*a* from the grid search and compared with the literature (Nuss *et al.*, 2010 ▸). An uncertainty of 0.003 follows the error analysis, step size and the uncertainty of the quadratic (Fig. 12 ▸, Table 3 ▸).

Paths 2 and 3 both include a component in the *z*-direction (along the *c*-axis) while path 1 does not. There is a progressive change downwards in α_3_ which could suggest a growing discrepancy in *c*/*a* or, for example, a discrepancy between the XAS and the XRD results. Both transmission and fluorescence confirm that the single-scattering path 1 is in agreement with XRD results (Nuss *et al.*, 2010 ▸), showing little change in lattice spacing in the *xy*-plane with temperature (Fig. 23 ▸).

σ_*j*_ has positive trends with temperature as expected from thermal behaviour, except for the 50 K dataset, which is compromised by the short range. Single-scattering paths which have a *z*-component are consistently larger than in-plane scattering paths, suggesting asymmetric dynamical motion within the crystal. σ_5_ is larger than σ_4_ since, on average, longer scattering paths means larger variance in the variation of the instantaneous path length from thermal motion. The energy offset is slightly large, though it is correlated with other parameters and the Hanning window. σ_4_ is smaller than other σ scattering paths noting that some of its major contributors are different permutations of scattering between the absorbing atom and nearest neighbours. Theoretical calculations with a cumulant expansion of the XAFS equation (Van Hung *et al.*, 2017 ▸) have predicted that the expansion coefficient should quickly go to zero below 100 K, and that σ^2^ should become steady in the range, as observed.

The minima in 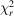 follow smooth quadratic curves (Fig. 12 ▸), yet show a consistent offset from the literature. The differences in *c*/*a* between this work and Nuss *et al.* (2010 ▸) in order of temperature are as follows. For the transmission data sets (T): |Δ(*c*/*a*)_T_| = 0.00695, 0.00630, 0.0107, 0.0132 and 0.00724; for the fluorescence data sets (F): |Δ(*c*/*a*)_F_| = 0.0140, 0.0143, 0.0117 and 0.0122. In terms of percentages these are 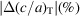 = 0.382%, 0.345%, 0.588%, 0.720% and 0.391%; 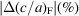 = 0.770%, 0.787%, 0.643% and 0.665%. XRD measures the distance between two atomic (lattice) sites in the unit cell, whereas XAFS measures the expectation value for the distance between two atoms at any instant. These can be summarized for any single pair of atoms as

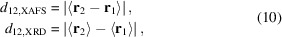

where **r**_1_ and **r**_2_ are the vector locations of two atoms and *d*_12_ is the scalar distance as defined either by XRD or XAFS. With any thermal motion for a single-scattering path we therefore expect *d*_12,XAFS_ ≥ *d*_12,XRD_ and the difference to be proportional to the magnitude of thermal vibrations. Extended cooling can lead to changes of structure or twinning, and hence a change of the contribution of static disorder, yet none were significant enough to be clearly observed.

## 8. Debye model

**Figure 24:**
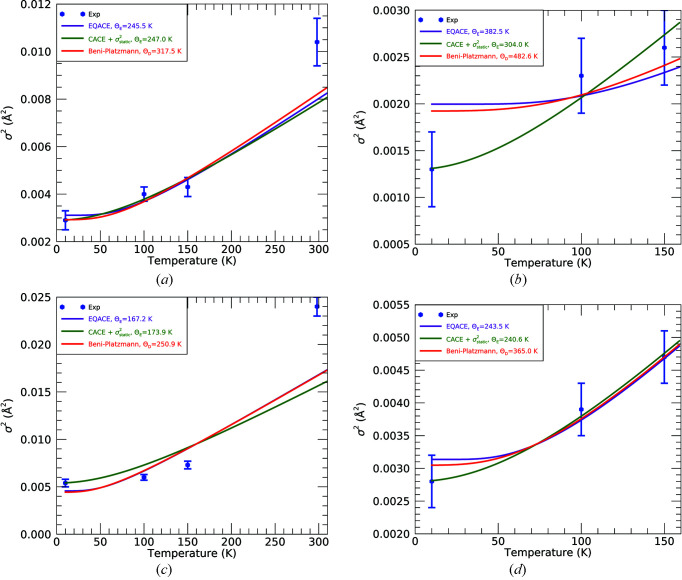
Comparisons of (*a*,*b*) first-shell and (*c*,*d*) third-shell Debye–Waller factors between experimental results for the EQACE, CACE and Beni–Platzmann (correlated Debye) models for the (*a*,*c*) transmission and (*b*,*d*) fluorescence fits.

Comparisons between first- and third-shell Debye–Waller factors ( 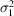 ) are shown in Fig. 24 ▸ in which the Debye and Einstein temperatures are fitted to the data. Here we compare with an extended quantum anharmonic correlated Einstein (EQACE) model (Tien, 2021 ▸) and with Beni–Platzmann theory (Beni & Platzman, 1976 ▸) which treat the vibrational density of states (VDOS) as a delta function at the Einstein frequency, and as a polynomial with a cut-off at the Debye frequency, respectively. We modify the classical anharmonic correlated Einstein (CACE) model which tends to 0 at 0 K, by adding in quadrature a static offset term, from σ^2^ at 10 K. We see good agreement with both the EQACE and Beni–Platzmann theories, which both return plausible results for their respective Einstein and Debye temperatures for the transmission data. The fluorescence data, however, demonstrate exceptionally poor agreement for the first shell with all theories returning unreasonably high values of Θ_E_ and Θ_D_. Our results do not immediately discriminate between these theories.

A key limitation of some approaches is the absence of any explicit static disorder term in both the EQACE and Beni–Platzmann models. Whilst our modified CACE model contains a static disorder term, the theory is not particularly robust below room temperature. None of these models fully represents the true VDOS of the system; however, the Beni–Platzmann (correlated Debye) model should be preferable. The characterization of dynamic bonding and thermal asymmetry and evolution is clearly observed in the data and modelling, and further work will characterize this more fully.

The precision and apparent accuracy of these results deserves some discussion. Early work (Crozier *et al.*, 1987 ▸) claimed to observe no significant evolution of parameters with temperature below 0.02 Å – that is, below 2 pm. Later work (Tröger *et al.*, 1994 ▸) claimed to determine nearest-neighbour bond distances to within 0.015 Å – that is, to within 1.5 pm. Exciting differential or relative XAFS measurements and their potential sensitivity to structure and function (Pettifer *et al.*, 2005 ▸) and sequels suggested the possibility of relative measurement of interatomic spacing evolution to 1 fm or 0.00001 Å using novel differential XAFS; which represented a differential Fourier transform in a non-conventional manner. The values presented therein achieved 0.01 Å or 1 pm and the authors commented that XAFS could, in principle, observe structure to 0.001 Å or 100 fm. They showed, however, that the noise floor could reach towards a femtometre scale.

Structural change hypotheses require a detailed assessment of uncertainty or significance as might follow from a 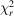 analysis or a Fischer test. In what follows, on the basis of careful measurement and assessment of systematic effects and corrections, we propagate uncertainties and achieve nearest-neighbour shell uncertainties of 0.0002–0.0004 Å or 0.02 pm (20 fm) for transmission with much bigger uncertainties for the limited 50 K dataset; and 0.0002–0.0020 Å or 0.02–0.20 pm for fluorescence. These are consistent with that level. We have measured and reported *c*/*a* values from different shell radii, and have assigned estimated uncertainties to these of 0.0015 or 0.030, corresponding to uncertainties of *r* of shells of order 0.3 pm or so. More importantly, we have compared this with the literature from XRD; part of the observed discrepancy is due to dynamical bond lengths versus site separation, so that our observed accuracy is approximately as stated. The advantage and advance is that data uncertainties were propagated, and that the improvement with standard XAS analysis is of the order of a factor of perhaps 30 compared with the very interesting differential XAFS work.

## 9. Supplementary information

Data reference and deposition are becoming very important in the XAS community. Hence it is highly recommended to have data available for other researchers on other beamlines and with other software for analysis. Hence we supply the four datasets for 10 K, 50 K, 100 K and 150 K in transmission in 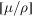 versus *E* in two formats suggested for cross-platform and reference work – an ifeffit-like .dat file and an IUCr-like .cif file. We supply the four datasets for 10 K, 50 K, 100 K and 150 K in fluorescence in 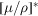 versus *E*. We supply the corresponding eight χ versus *k* data sets for processing. We additionally supply the 298 K χ versus *k* data set for processing.

The files labelled Zn-murho-(10-150)K-transmission.cif include the information on the data collection together with seven columns containing the energy, energy error, attenuation coefficient 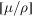 , absolute error in the attenuation coefficient 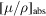 , relative error in the attenuation coefficient 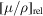 , photoelectric attenuation coefficient 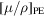 and the relative error in the photoelectric attenuation coefficient 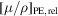 post-systematic correction from the transmission mode. Files labelled Zn_murho_(10150)K_fluroescence.dat contain three columns detailing the energy, attenuation coefficients and their associated relative error post-systematic and self-absorption correction from the fluorescence mode. The files labelled as Zn_chi_k_(10150)K_transmission.dat and Zn_chi_k_(10150)K_fluorescence.dat contain the *mu2chi* output of each of the transmission and fluorescence files, respectively, and contain the *k*, χ, Δχ and *E*_0_ values that can be fed directly into *efeffit* for analysis. Finally the file Zn_chi_k_298K_transmission.dat also contains the post-*mu2chi* data from Ekanayake *et al.* (2021*a* ▸,*b* ▸) which we compare directly with in this work; the original attenuation data files can be found in the supplementary information section of those papers. That is, a total of 17 data sets are supplied in the supporting information. Headers explain columns and format.

## 10. Conclusion

We report the first transition metal XAFS using the hybrid technique and at low temperatures. This is also the first (hybrid-like) experiment at the Australian Synchrotron. We report high-accuracy measurement of the XAS of zinc metal at the Zn *K*-edge. We report a methodology for cryogenic and temperature series measurements which is robust, valid for both transmission and fluorescence XAS, and attains parameter accuracy. We demonstrate the use of *SeAFFluX* as a precision tool for converting fluorescent spectra to extract the absorption XAFS on an absolute scale. We observed thermal evolution and dynamic lattice parameter ratios in very good agreement with literature XRD values, noting the expected difference between dynamic bonding (XAFS) and site separation (XRD). We are able to investigate aspects of thermal behaviour models using this approach. More work is called for.

## Supplementary Material

Full data sets in cif-like and text formats. DOI: 10.1107/S1600577522010293/rv5168sup1.zip

## Figures and Tables

**Figure 20:**
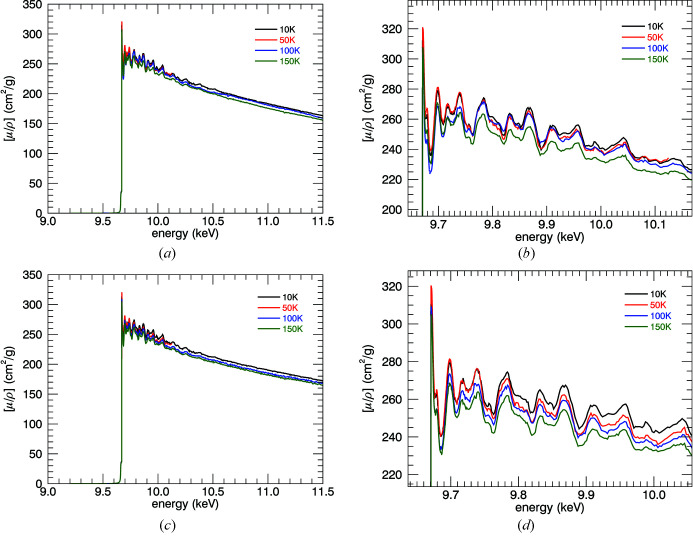
Results presented as a temperature series for the (*a*, *b*) 10 µm and (*c*, *d*) 50 µm foils. These share a very strong correlation with our attenuation results.

**Figure 25:**
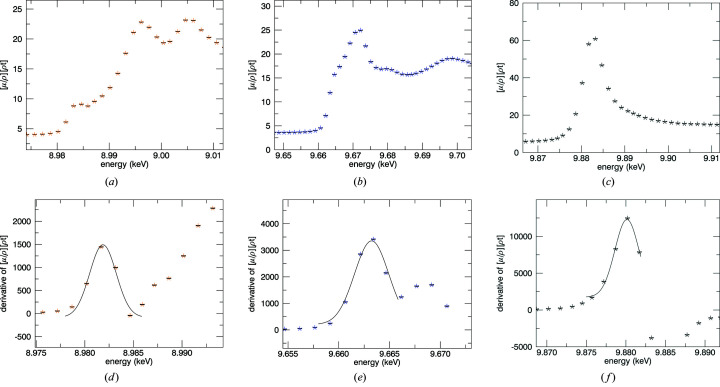
(*a*)–(*c*) Cu *K*, Zn *K*, Ta *L*_3_ edges and (*d*)–(*f*) fits of the derivative peak. Uncertainties are 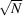 counts assuming a Poisson distribution.

**Figure 26:**
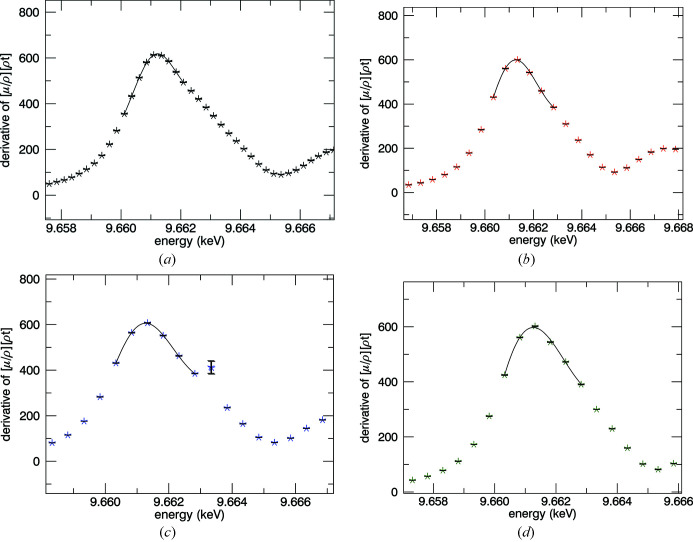
Determination of the Zn *K* absorption edge by finding the first peak in the derivative spectra for (*a*) 10 K, (*b*) 50 K, (*c*) 100 K and (*d*) 150 K. The energy is determined to a fraction of the point spacing.

**Figure 27:**
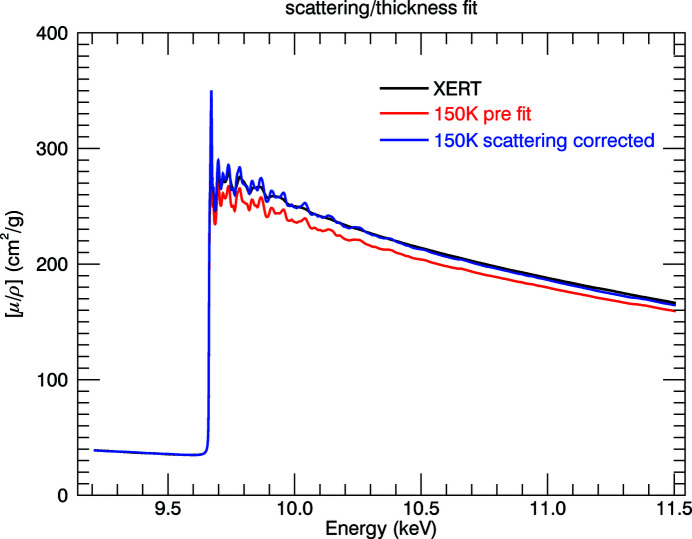
Effect of correcting for the scattering according to equation (13). The model is effective across the whole energy range. ‘XERT’ is the reference data taken at 298 K (Ekanayake *et al.*, 2021*a* ▸,*b* ▸; Sier *et al.*, 2022 ▸). ‘150 K’ is this work, for *T* = 150*K*.

**Table 1:** Dark current dependence upon time The dark-current measurements in Fig. 4 ▸ show three distinct regions: 15–30 h; 30–50 h; remainder. 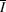 represents the total counts at each ion chamber while σ_sd_ and σ_se_ are the standard deviation and standard error, respectively. In the first region of ion chambers *I*_1_ and *I*_2_, a linear model [*D*_*i*_ = *A* + *B*(*t* − *t*_0_)] is applied.

	*I*_0_			*I*_1_			*I*_2_		
Region	1	2	3	1	2	3	1	2	3
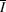	1873.20	1856.44	1857.43	2063.83	2052.91	2059.52	1323,80	1312.45	1318.30
σ_sd_	1.60	1.92	1.54	2.53	1.91	1.76	1.16	1.95	1.30
σ_se_	0.073	0.075	0.336	0.115	0.074	0.383	0.053	0.076	0.283
*A*				2063.87			1323.85		
*B* (h^−1^)				0.108			0.13		
*t*_0_ (h)				25			25		

**Table 2:** Contributions to the overall uncertainty from each source Corrections are labelled 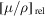 if they contribute to the shape of the spectra with an independent contribution to uncertainty at each point. 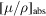 notes corrections that relate to broad scale corrections such as the material thickness. The table gives the maximum individual uncertainty and the average contribution 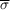 . Extra sources of systematic and uncertainty arising from bandwidth and nanoroughness cannot be measured with a single foil in the current setup.

Quantity	Magnitude of correction [μ/ρ] (cm^2^ g^−1^)	Percent uncertainty standard error estimate, σ_[μ/ρ]_	Origin or cause
		<0.075%	Counting variance†
[μ/ρ]_rel_	1.16% to 9.20%	<0.014%	Dark current‡
	−52.90% to 3.84%	<0.282%	Blank normalization§

[μ/ρ]_abs_	11.38%	0.024%	Thickness¶

[μ/ρ]_rel_	α < 5 × 10^−5^	0.0001% to 0.01%	Harmonic correction††
	3.2% to 11.4%	0.01% to 0.19%	Scattering‡‡
	0.14% to 1.54%	0.028% to 0.29%	Roughness§§
		0.024% to 0.23%	Absolute uncertainty¶¶
		0.039%	Final average σ

*E* (keV)	0.5 eV	<0.1 eV	Energy†††

† Standard error from counting statistics.

‡ Dark-current correction and corresponding uncertainty.

§ Blank current correction and corresponding uncertainty.

¶ Uncertainty from thickness correction (Ekanayake *et al.*, 2021*b*), see Appendix *C* [App appc].

†† Harmonic coefficient and contribution is negligible.

‡‡ Secondary photons from fluorescent scattering. Correction maximal directly above absorption edges, 0 below the Zn edge. Contribution from scattering, equation (14).

§§ Roughness of thin sample; bandwidth from beam footprint on sample. See Appendix *E* [App appe].

¶¶ Uncertainty in [μ/ρ] after all corrections. See Appendices *A* [App appa]–*E* [App appe].

††† Uncertainty in energy calibration. See Appendix *B* [App appb].

**Table 3:** *eFEFFit* fits α_1,2,3_ and 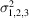 were assigned to the three nearest-neighbour single-scattering paths. α_4_ and 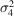 were assigned to other paths out to *r*_eff_ = 5.32 Å, and the rest of the paths out to *r*_eff_ = 7 Å were assigned α_5_ and 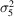 . *r*_1,2,3_ are the three nearest-neighbour atomic distances defined by the respective α. See text.

	10 K	50 K†	100 K	150 K	298 K
*c*/*a*	1.819	1.824	1.825	1.828	1.849
*c*/*a*_Nuss_	1.826†	1.830	1.836	1.841	1.857
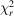	25.0	57.3	15.0	11.9	29.1
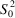 ‡	1.0	1.0	1.0	1.0	1.0
Δ*E*_0_ (eV)	4.9 (2)	4.5 (5)	4.5 (2)	4.6 (2)	−5.7 (7)

α_1_	1.0022 (8)	1.005 (1)	0.9996 (8)	0.9994 (7)	1.010 (1)
α_2_	1.003 (1)	1.009 (2)	1.00001 (91)	1.0012 (9)	1.141 (3)
α_3_	0.995 (1)	0.9995 (1)	0.998 (1)	0.997 (1)	1.031 (4)
α_4_	0.9976 (6)	0.999 (1)	0.9984 (6)	0.9985 (6)	1.021 (2)
α_5_	0.994 (1)	0.998 (2)	0.996 (1)	0.995 (1)	1.036 (2)

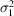 (Å^2^)	0.0029 (1)	0.0003 (3)	0.0040 (1)	0.0043 (1)	0.0104 (5)
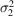 (Å^2^)	0.0070 (3)	0.0042 (7)	0.0056 (2)	0.0071 (2)	0.022 (1)
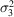 (Å^2^)	0.0054 (4)	0.0019 (6)	0.0060 (3)	0.0073 (4)	0.024 (1)
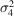 (Å^2^)	0.0040 (2)	0.0015 (4)	0.0052 (2)	0.0058 (2)	0.020 (1)
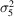 (Å^2^)	0.0054 (6)	0.004 (1)	0.0081 (7)	0.0091 (7)	0.021 (1)

*r*_1_ (Å)	2.6657 (2)	2.674 (4)	2.6589 (2)	2.6737 (2)	2.688 (4)
*r*_2_ (Å)	2.8731 (3)	2.897 (6)	2.8723 (2)	2.9019 (2)	3.309 (9)
*r*_3_ (Å)	3.8912 (4)	3.912 (7)	3.9079 (4)	3.9153 (4)	4.056 (1)

† The 50 K data were collected with a much narrower *k*-range, up to *k* = 11 Å^−1^.

‡ *c* = *a* from Nuss *et al.* (2010) only included experimental values down to 50 K, hence this is a linearly extrapolated value.

§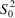 would drift above 1.15 to attempt to accommodate the extra sharp peaks. Hence it was fixed at 1; the fitting compensated by reducing σ^2^ broadening.

**Table 4:** Fitted parameters using fluorescent data sets; defined as for the transmission measurements, Table 3 ▸

	10 K	50 K	100 K	150 K
*c*/*a*	1.812	1.816	1.824	1.829
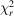	9.59	15.60	6.33	4.75
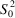	1.0	1.0	1.0	1.0
Δ*E*_0_	4.0 (3)	4.4 (5)	4.0 (3)	3.4 (3)

α_1_	1.0045 (9)	1.006 (1)	1.002 (1)	1.0042 (9)
α_2_	1.006 (1)	1.010 (2)	1.004 (1)	1.009 (1)
α_3_	0.998 (1)	0.998 (2)	0.9994 (13)	1.001 (1)
α_4_	0.9989 (7)	0.998 (1)	0.9998 (7)	1.0012 (7)
α_5_	0.995 (1)	0.995 (2)	0.997 (1)	0.998 (1)

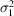 (Å^2^)	0.0013 (1)	0.0004 (4)	0.0023 (1)	0.0026 (1)
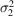 (Å^2^)	0.0070 (5)	0.0040 (8)	0.0041 (3)	0.0062 (3)
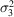 (Å^2^)	0.0028 (4)	0.0027 (7)	0.0039 (4)	0.0047 (4)
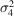 (Å^2^)	0.0018 (2)	0.0017 (4)	0.0029 (2)	0.0035 (2)
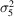 (Å^2^)	0.0043 (8)	0.004 (1)	0.007 (1)	0.008 (1)

*r*_1_ (Å)	2.6721 (2)	2.675 (4)	2.665 (2)	2.675 (2)
*r*_2_ (Å)	2.8755 (4)	2.890 (7)	2.883 (3)	2.905 (3)
*r*_3_ (Å)	3.8951 (5)	3.898 (8)	3.912 (5)	3.909 (5)

**Table 5:** Determination of the absorption edge by finding the first peak in the derivative spectra Each temperature is self-consistent to within error and demonstrates a potential calibration error of 0.5 eV.

	Derivative peak	Literature	Difference	χ^2^
Zn 10 K	9661.23 (7)	9660.755 (30)†	+0.47	0.037
Zn 50 K	9661.30 (3)	9660.755 (30)†	+0.54	0.32
Zn 100 K	9661.28 (6)	9660.755 (30)†	+0.52	0.061
Zn 150 K	9661.29 (6)	9660.755 (30)†	+0.53	0.037

† Kraft *et al.* (1996 ▸).

**Table 6:** Results from fitting the reference foils are inconsistent – reference and deviations do not follow a physical progression with energy

	Derivative peak	Reference	Difference	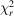
Cu *K*	8981.87 (21)	8980.476 (20)†	+1.39	2.7 × 10^4^
Zn *K*	9663.23 (18)	9660.755 (30)†	+2.47	1.2 × 10^5^
Ta *L*	9880.13 (10)	9881.1‡	−1.00	3.42 × 10^5^

† Kraft *et al.* (1996 ▸).

‡ Wong (1999 ▸).

## Appendix A Harmonic contributions

The equipment was mounted at a beamline which did not have a pre-mirror between the source and the double-crystal monochromator. Such mirrors are frequently used as a high cut filter to eliminate high-energy radiation which might interfere with the experiment. A Si(111) double-crystal monochromator was used in this experiment. The Si(222) reflection is forbidden; so the major contributor to harmonics is the Si(333) reflection at an energy three times that of the fundamental energy.

Since the third-order harmonics in general have a much lower attenuation coefficient, the linear relationship between thickness *t* and absorption  is not valid. Thicker foils will absorb most of the fundamental energy photons and higher-energy photons will then dominate in the downstream beam. Quantifying this non-linearity gives

where α is the proportion of harmonic content, the effective harmonic content (Tran *et al.*, 2003 ▸; de Jonge *et al.*, 2005 ▸; Glover & Chantler, 2009 ▸), [μ/ρ]_f_ is the mass attenuation coefficient at the fundamental energy, and [μ/ρ]_h_ the mass attenuation coefficient at the harmonic energy. An extra parameter DC_offset_ is added to check the robustness and accuracy of the earlier determination of dark-current measurements – it is zero within uncertainty. Normalization of the local daisy wheel foil thicknesses in the beam is performed at the highest energy, where harmonic contamination is considered negligible; in this case 11.29 keV (Tran *et al.*, 2003 ▸). Nine aluminium foils in the daisy wheels were used. Two independent methods may be used to determine the daisy wheel foil thicknesses, relying on either the nominal thicknesses or *FFAST* attenuation tables:

(1) The first uses the experimentally derived  =  and mass attenuation coefficients [μ/ρ] from *FFAST* (Chantler, 1995 ▸, 2000 ▸). The ratio between these two, divided by the known density, retrieves the thickness.

(2) The second plots measure  =  against [ρ*t*] from nominal thickness values and then fits a straight line to determine [μ/ρ]. Then, ρ*t* is determined with fitted values of [μ/ρ].

The two methods differed by <10^−5^%. Harmonic measurements were taken at various instances during the experiment, spanning the whole energy range. The results presented limit the dark-current offset to about one standard deviation as in Table 1 ▸.

With most energy points returning α indistinguishable from 0 and others below α = 5 × 10^−6^, and given that the intensity downstream with no foils is between 2 × 10^5^ and 3 × 10^5^ counts, this equates to a maximum contamination of 1–2 counts at each point. The dark-current standard deviation is ∼1.5–2.5 counts in the upstream and downstream ion chambers. By substituting a value for the effective harmonic parameter of 5 × 10^−6^ into equation (11), we observe that any harmonic contamination will be negligible. Hence we were able to investigate this carefully, but it was not a major source of systematic error in this experiment.

## Appendix B Energy calibration

Hysteresis in the positioning motors of the monochromator can cause the energy to drift over large energy ranges; hence an independent energy measurement is desired. Standard XAFS uses a single calibrated reference foil. Used in its original form, XERT should directly measure the energy of the beam through the sample with an independent powder or crystal reference. In the absence of this at this beamline, we used zinc, copper and tantalum reference foils (Fig. 25 ▸). The edge energy in a material is most commonly defined experimentally as the first peak in the derivative spectra (Kraft *et al.*, 1996 ▸). Energy resolution and photon bandwidth affect this energy in an ill-defined manner. Using this definition, the Zn *K*-edge was 9663.23 (18) eV, the Cu *K*-edge was 8981.87 (21) eV and the Ta *L*_3_-edge was 9880.13 (10) eV, compared with reference values of 9660.755 (30) eV, 8980.476 (20) eV (Kraft *et al.*, 1996 ▸) and 9881.1 eV (Wong, 1999 ▸).^1^

The zinc *K*-edge energy is also determined at the four temperatures from the collected data sets, which have a much finer point spacing than the reference foil sets.

In Fig. 26 ▸ a Gaussian has been fitted with a linear background to deal with asymmetry. Derivative peak values ranged from 9661.23 eV to 9.661.30 eV with standard deviations <0.1 eV, which is a consistent offset from the value 9660.755 (30) eV (Kraft *et al.*, 1996 ▸) of 0.5 eV. The consistency for all temperatures suggests that the monochromator was consistent over time, and *may* correspond to a single energy offset of 0.5 eV (Table 5 ▸). This correction is important and useful, but not that definitive and subject to further investigation in the future, especially given the mixed evidence provided by Table 6 ▸. However, one can argue that the XAFS is simply offset by this amount and that XAFS fitting software is therefore somewhat independent of this correction.

## Appendix C Thickness determination

To convert from  to  (cm^2^ g^−1^) requires a precise measurement of the foil thickness at the point where it interacts with the X-ray beam; or scaling to a calibrated reference. A recent experiment on room-temperature zinc foils made an accurate measurement of foil thickness allowing scaling of these  spectra (Sier *et al.*, 2022 ▸; Ekanayake *et al.*, 2021*a* ▸,*b* ▸). The column density was determined by selecting a small well defined energy range in the pre-edge spectra of the 150 K and room-temperature 298 K data,

The scaling factor calculated was 87.372 cm^2^ g^−1^ for a density of 7.112 g cm^−3^. The foil is at 45° so corresponds to a foil thickness of 11.380 µm which is within the manufacturer specifications of 10 µm ± 15%. The accuracy in thickness claimed in the room-temperature series is <0.024%. Measurements of thermal expansion with temperature using XRD have some uncertain error bars (Nuss *et al.*, 2010 ▸). The *c*/*a* ratio is the dominant variation; from 10 K to 150 K this is 5.5 × 10^−5^ K^−1^; from 150 K to 298 K it is roughly 6.2 × 10^−5^ K^−1^, fairly uniform and monotonic. Theoretical calculations suggest that the thermal expansion via a cumulant approach should go rapidly to zero below 100 K; similarly, experimental XRD results (Nuss *et al.*, 2010 ▸) suggest an expansion coefficient below 160 K of 0 K^−1^ and between 160 K and 500 K of 0.0033 pm K^−1^ or 1.2 × 10^−5^ K^−1^; or 1.2 × 10^−3^% K^−1^ in the *a* = *b* axes; and 1.2 × 10^−4^ K^−1^; or 12 × 10^−3^% K^−1^ in the *c* axis. [ρ*t*] (g cm^−2^) is the integrated column density. Our results below are consistent with this; hence any change of the integrated column density in the beam cross-section is orientationally dependent, but cancels in the *c* plane. The density increases by a small amount with decreasing temperature, but the thickness also decreases; hence to first order the total integrated column density remains constant. The same scaling factor was then applied to all temperatures so as to preserve the inherent small variations due to temperature. This correction is crucial for a reference spectrum as a function of temperature. Yet most XAFS software will scale the values. Hence it is particularly important if there are any energy-dependent functionalities above the edge.

## Appendix D Scattering and fluorescence

*K*α fluorescence or scattered photons from a sample can travel backwards or forwards into either the upstream or downstream ion chambers. XERT has daisy wheels between the sample and each ion chamber with various aperture sizes. Since the photon source from the synchrotron is collimated and much narrower than the apertures, variations in counts due to changing apertures are dominated by fluorescence and Rayleigh scattered photons (because the Compton inelastic scattering component is peaked at 90° to the beam and sample).

Previous work with zinc at the same beamline measured the scattering and fluorescence contributions in each ion chamber (Sier *et al.*, 2020 ▸; Trevorah *et al.*, 2019 ▸; Islam *et al.*, 2015 ▸). Hence we require a functional to match the scattering correction of each data set. In a hybrid setup, the counts in a fluorescence detector should be directly proportional to fluorescence photons reaching the ion chambers, with that proportionality determined by relative aperture sizes and detector efficiencies. A correction functional may be used,

*A* = 1/[ρ*t*] from the thickness determination, *f* is the fluorescence counts and α, β are constants to be fitted. α and β define the fraction of fluorescence which enters the upstream and downstream (fluorescence) detectors (Sier *et al.*, 2020 ▸; Trevorah *et al.*, 2019 ▸; Islam *et al.*, 2015 ▸).

Upon inspection, any scattering is below the noise level in the upstream ion chamber – hence α is set to zero, with β particularly fitted from the high-energy *E* or high *k*-range. The model correctly matches previously calibrated data (Fig. 27 ▸). Ideally a full data set of transmission and fluorescence can characterize this and allow inversion from the data, either with *SeaFFluX* software or with simulation modelling. However, the additional source of systematic error and uncertainty generated by the correction can be incorporated by looking at the effect on the ion chamber ratio with standard error uncertainties (with α = 0),

using the intermediate variable σ_ln_,

From Fig. 27 ▸, the correction is important for the absolute value of photoabsorption and for the energy dependence of the structural correction. This has been applied to the data.

## Appendix E Roughness and bandwidth

The nanoroughness of the sample is a change in the integrated column density [ρ*t*] through the beam footprint, including surface roughness and internal roughness. It affects the accuracy, especially for thin foils like those we are using (de Jonge *et al.*, 2007 ▸). The change in the mass attenuation coefficient due to nanoroughness is

where [μρ] is the measured mass attenuation coefficient, [ρ*t*]_av_ is the average integrated column density and σ_[ρ*t*]_ is a roughness distribution parameter. As we do not have measurements conducted over a range of different sample thicknesses, we cannot independently investigate the sample nanoroughness with these data. Given that this experiment used the same sample foil as Ekanayake *et al.* (2021*b* ▸), we take the value obtained in that analysis, σ_[ρ*t*]_ = (9.9 × 10^−4^ ± 4 × 10^−6^) g cm^−2^ for the 10 µm sample, with a 10% uncertainty. This results in a correction of the data of up to 2.52% for the 10 µm sample.

The effect of the energy bandwidth in the incoming X-rays, on the spectra, should also be considered. The Si(111) monochromator allows for a small bandwidth of energies that are centred around our desired *E*_0_ due to intrinsic bandwidth, heat load and thermal and mechanical stresses within the monochromator. The absorption can be modelled by

where *I* represents the intensity around an infinitesimal energy range d*E* centred on *E*_0_ and  is the measured mass attenuation coefficient at nominal energy. The effect of this will be seen predominantly as a broadening in areas of high absorption gradient such as an absorption edge, and should also grow with sample thickness.

With only a single foil thickness, we can take a similar approach as with the nanoroughness, and compare with earlier calibrated data (Ekanayake *et al.*, 2021*a* ▸,*b* ▸) to observe the signature. In comparing two temperatures (150 K and room temperature), we expect to see some thermal broadening. By looking at the sharp first edge of the peak, we see where the thermal broadening is at its lowest, and the bandwidth effect is at its highest. Still in this range, the thermal broadening is dominant with no evidence of further effects from bandwidth, hence we can progress confidently without significant bandwidth effects.
